# Resource recovery and biochar characteristics from full-scale faecal sludge treatment and co-treatment with agricultural waste

**DOI:** 10.1016/j.watres.2019.115253

**Published:** 2020-02-01

**Authors:** Benedict C. Krueger, Geoffrey D. Fowler, Michael R. Templeton, Berta Moya

**Affiliations:** a Department of Civil and Environmental Engineering, Imperial College London, SW7 2AZ, UK; b Biomass Controls LLC, Putnam, CT, USA

**Keywords:** Faecal sludge, Biochar, Co-treatment, Sanitation

## Abstract

Unsafe disposal of faecal sludge from onsite sanitation in low-income countries has detrimental effects on public health and the environment. The production of biochar from faecal sludge offers complete destruction of pathogens and a value-added treatment product. To date, research has been limited to the laboratory. This study evaluates the biochars produced from the co-treatment of faecal sludge from septic tanks and agricultural waste at two full-scale treatment plants in India by determining their physical and chemical properties to establish their potential applications. The process yielded macroporous, powdery biochars that can be utilised for soil amendment or energy recovery. Average calorific values reaching 14.9 MJ/kg suggest use as solid fuel, but are limited by a high ash content. Phosphorus and potassium are enriched in the biochar but their concentrations are restricted by the nutrient-depleted nature of septic tank faecal sludge. High concentrations of calcium and magnesium led to a liming potential of up to 20.1% calcium carbonate equivalents, indicating suitability for use on acidic soils. Heavy metals present in faecal sludge were concentrated in the biochar and compliance for soil application will depend on local regulations. Nevertheless, heavy metal mobility was considerably reduced, especially for Cu and Zn, by 51.2–65.2% and 48.6–59.6% respectively. Co-treatment of faecal sludge with other carbon-rich waste streams can be used to influence desired biochar properties. In this case, the addition of agricultural waste increased nutrient and fixed carbon concentrations, as well as providing an additional source of energy. This study is a proof of concept for biochar production achieving full-scale faecal sludge treatment. The findings will help inform appropriate use of the treatment products as this technology becomes more commonly applied.

## Highlights

• First comprehensive study on full-scale faecal sludge treatment producing biochar.
• Biochar properties suggest potential applications as solid fuel or soil amendment.
• Complete destruction of pathogens and partial immobilisation of heavy metals.
• Co-processing with agricultural waste improves biochar properties.

## Graphical abstract

**Figure:**
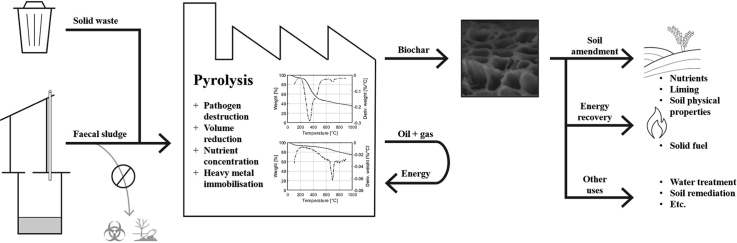

## 1 Introduction

It is estimated that 4.5 billion people are not using safely managed sanitation services (UNICEF and WHO, 2017). Approximately 2.7 billion people rely on pit latrines, septic tanks or other onsite sanitation facilities (Strande et al., 2014). Uncontrolled disposal of untreated faecal sludge (FS) from such systems to local water bodies is detrimental to both the environment and public health. Compared to municipal wastewater, FS typically contains more than ten times the amount of organic and pathogenic contamination (Ingallinella et al., 2002). Finding workable solutions for FS treatment that offer a viable alternative to illegal dumping is therefore crucial.

Recent research has investigated various thermochemical treatment technologies for FS treatment including combustion (Gold et al., 2017; Onabanjo et al., 2016), gasification (Recalde et al., 2018) and hydrothermal carbonisation (Fakkaew et al., 2018). In particular, the feasibility of biochar production for FS treatment has been suggested (Bond et al., 2018) and the suitability of FS pyrolysis for solid fuel production assessed (Andriessen et al., 2019).

**Fig. 1:**
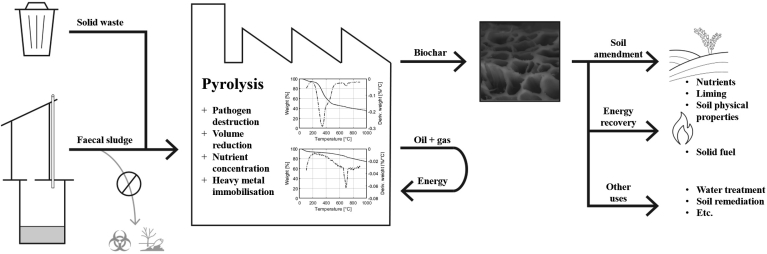
Conceptual diagram of biochar production for faecal sludge treatment.

Biochar is a charcoal-like material that is obtained through a process called pyrolysis, where an organic feedstock is heated in an oxygen-limited or oxygen-free environment at temperatures between 350 and 800^∘^C. Biochars are produced for environmental applications such as soil improvement or to aid in carbon sequestration. However, pyrolysis is also an established method in waste management, including the treatment of sludge and manure (Joseph and Lehmann, 2015; Smith et al., 2009). Fig. 1 outlines a concept for integrating this technology into the management of FS. Experimental studies have examined the characteristics of FS-derived biochars as a soil amendment (Woldetsadik et al., 2018), solid fuel (Ward et al., 2014) and adsorbent (Koetlisi and Muchaonyerwa, 2017) or have assessed different uses simultaneously (Gold et al., 2018; Liu et al., 2014). FS pyrolysis has also been the subject of thermodynamic modelling (Yacob et al., 2018).

Table 1 provides an overview of advantages and disadvantages associated with biochar production from FS. Compared to other biological or physical treatment methods, the short retention time of a continuous pyrolysis process allows for minimal space requirements. The paucity of FS pyrolysis research is still a key drawback for this technology, especially as the production of biochar in developing countries has been negatively connotated as a cause of air pollution (Lohri et al., 2016). In thermal treatment processes, including FS pyrolysis, feedstock with high water content drastically reduces energy efficiency (Bond et al., 2018). However, the option to co-treat other carbonaceous waste fractions, even plastics, can improve the energy balance (Ro et al., 2014). This option to co-treat FS for biochar production is missing from previous experimental studies. Considering that organic waste and low-grade plastics, both sources of carbon, are a main concern in the waste management of low- and middle-income countries (Wilson and Webster, 2018), co-treatment should be considered.

**Table 1:** Advantages and disadvantages of biochar production as FS treatment.

Advantages	Disadvantages
• Complete destruction of pathogens • Low space requirement for continuous processes • Net energy surplus depending on FS water content • Co-treatment with other waste fractions • Containerised design possible • Storage, transport and disposal of outputs simplified through reduced volume and biochemical stability • Biochar applications including soil amendment, fuel, adsorption media and C sequestration • Phosphorus and potassium are retained in biochar • Biochar may improve physiochemical soil properties	• Limited availability of research data • Little to no experience at scale • Relatively complex process control • High operational temperatures • Energy-intensive drying required before pyrolysis • Need for flue gas treatment • Inadequate design may lead to harmful air emissions • Nitrogen is largely lost to the vapour phase • Potential formation of organic contaminants in biochars such as polycyclic aromatic hydrocarbons (PAHs), dioxins and furans

FS pyrolysis offers complete destruction of pathogens due to high processing temperatures (Ward et al., 2014). This is a key advantage for the agricultural use of FS-derived biochars, adding to the value from any plant nutrients retained in the biochar. Gold et al. (2018) and Woldetsadik et al. (2018) investigate the supply of such nutrients from FS biochars, but do not assess the potential to alleviate acidic soil conditions as observed for biochars produced from animal manure (Singh et al., 2017). Gold et al. (2018) found FS biochars to breach the total heavy metal concentration limits of international biochar guidelines. While this is a main concern for any soil application there is also a need to understand how pyrolysis influences the fate of heavy metals in the environment. A reduction in the mobility of heavy metals has been reported in biochars produced from contaminated soil (Wijesekara et al., 2007) and should therefore also be considered for FS.

Previously, research on FS biochars has been at laboratory scale only. There is a necessity to demonstrate FS treatment technologies for resource recovery at full scale (Andriessen et al., 2019; Diener et al., 2014) and, in light of the sanitation crisis, for scaling to occur rapidly (Strande et al., 2014). Therefore, the objective of this study was to evaluate the biochar produced from the first FS treatment plants of their kind operating at full-scale in India. This encompassed the characterisation of the precursor material, its thermal degradation, the resulting biochar properties and their applicability as a soil amendment or solid fuel.

## 2 Materials and methods

### 2.1 Sampling

Warangal Faecal Sludge Treatment Plant: 17°56’15.5"N 79°33’31.9"E.

Narsapur Faecal Sludge Treatment Plant:16°25’38.2"N 81°42’01.2"E.

The biochar samples were taken at the FS treatment plants in Warangal, Telangana and Narsapur, Andhra Pradesh, India in October 2018. Both plants receive FS collected from septic tanks in the local area. Before treatment the FS is stored in holding tanks allowing for homogenisation of its characteristics, which vary considerably between individual containments (Strande et al., 2018). It is then dewatered and thermally dried. The FS is co-treated with pellet fuel (PF) derived from agricultural waste (0.3  kg PF/kg FS dry basis). In India, agricultural waste is the most frequently used raw material for PF production (Purohit and Chaturvedi, 2018). Such PF is locally produced and sold, but the product does not specify the origin of the agricultural residues. The composition will most probably be influenced by the regional and seasonal crops which are available.

Sampling was conducted by incremental cross-stream sampling, as suggested for continuously produced biochar (Singh et al., 2017). Three samples of biochar were collected daily over a four-day period, resulting in twelve samples from Warangal (W-BC) and twelve from Narsapur (N-BC). During this period a composite sample of dewatered FS was collected and thermally dried for feedstock characterisation at both Warangal (W-FS) and Narsapur (N-FS). Two batches of control biochar (CBC) using only solar-dried FS, which was fed directly to the reactor without PF, were produced both at Warangal (W-CBC) and Narsapur (N-CBC). Solar-drying was conducted on-site for a 10 h period and facilitated by spreading the sludge in a thin layer of approximately 10 mm depth. The control batches allow an assessment of the effects on biochar properties from co-treated FS and PF compared to the production exclusively from FS.

### 2.2 Process configuration

**Fig. 2:**
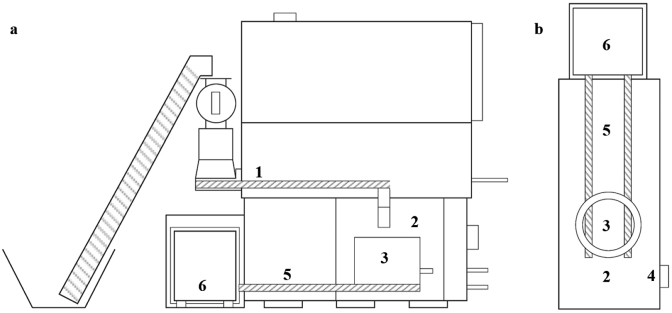
Long section (a) and plan view (b) of the reactor design.

Fig. 2 shows the process configuration of the Biomass Controls Biogenic Refinery Model 4018 (Biomass Controls LLC, USA) operational in Warangal and Narsapur. The process is specified to treat 360 kg FS/day (dry basis). A feedstock auger (1) transfers the dewatered and thermally-dried FS and PF mixture to the reactor inlet above the main reaction chamber (2), where it is delivered into the reaction vessel (3). Heat from the main reaction chamber will cause thermal degradation to start in the feedstock auger. The chamber receives a limited supply of oxygen through an air fan (4) to allow for partial oxidation enabling autothermal operation. The material migrates downwards through the reaction vessel and is removed by two char augers (5) at the base of the vessel, which convey it to a collection box (6).

### 2.3 Analytical methods

The biochar particle size class was determined on site by progressive dry sieving with 500 μm, 1000 μm and 2000 μm mesh sizes (Schmidt et al., 2015). All samples were subsequently manually ground, sieved to <500 μm and dried to constant weight. Samples were analysed in triplicate and are reported as mean values.

The pH was measured by Standard Method 9045D (USEPA, 2004) adjusting the sample-to-water ratio to 1:10 and the mixing period to 1 h as recommended for biochar (Singh et al., 2017). CaCO_3_-equivalents (CCE) were determined by adding standardised 0.5M HCl to the sample in a 1:20 solid-liquid ratio, mixing for 2 h followed by a 16-h resting period. The suspension was then titrated to pH 7 with 0.25M NaOH (Singh et al., 2017).

To analyse the inorganic composition of the samples, 0.2 g of sample was dry-ashed at 500^∘^C according to Standard Method 3030J (APHA, 1992). The samples were then digested at 95^∘^C for 2 h according to Standard Method 3050B (USEPA, 1996). To provide a stronger digestion, aqua regia was used instead of nitric acid. The addition of hydrogen peroxide was omitted due to the preceding dry-ashing cycle. The mobility of metals in the sample was determined by extraction in a 20:1 liquid-solid ratio according to the Toxicity Standard Leaching Procedure (TCLP), Standard Method 1311 (USEPA, 1992). The extractant used for this procedure was 1M HCl to simultaneously determine the plant-available fraction of Potassium (K_*available*_) in biochars (Singh et al., 2017). Plant-available phosphorus (P_*available*_) was determined by 2% formic acid extraction (Wang et al., 2012). The analytes from the digestion and extractions were filtered through Whatman No. 41 filter paper and analysed by ICP-OES (Avio 500,PerkinElmer, USA). Quality control complied with the spike recovery requirements of Standard Method 6010D for ICP-OES analysis (USEPA, 2018).

The calorific values were determined by bomb calorimetry (Parr 6100 calorimeter). Proximate and thermal analysis was conducted on a PL Thermal Sciences Simultaneous Thermal Analyser 1500 (PL-STA 1500). Volatile matter, fixed carbon and ash content were determined in an automated procedure comprising a drying phase at 105^∘^C and continuous heating to 950^∘^C in an inert nitrogen atmosphere, followed by cooling and then combustion in air at 600^∘^C. Curves for thermogravimetric analysis (TGA) and derivative thermogravimetry (DTG) were obtained by gradually heating (10^∘^C/min) the sample to 1000^∘^C under a flowing nitrogen atmosphere and recording the weight change for interpretation. Thermal analysis was conducted for PF and FS to analyse the feedstock’s thermal degradation, and for CBC to assess the effectiveness of the reactor in achieving full pyrolysis of the feedstock.

Ultimate analysis for CHNS was performed by MEDAC Ltd., Surrey, UK. SEM images were taken with a TM4000 Tabletop Microscope (Hitachi, Japan).

### 2.4 Heavy metal immobilisation

The control samples (W-CBC and N-CBC) were assessed to quantify any changes in the mobility of heavy metals, thus eliminating any inaccuracies stemming from heavy metal contamination through the PF. It also avoided errors in the mass balance associated with the approximate mixing of PF and FS by the operators. Chromium (Cr), copper (Cu), manganese (Mn), nickel (Ni) and zinc (Zn) were selected, as they have been reported as non-labile; they largely remain in the solid residue of sewage sludge and solid waste thermal treatment processes (Chanaka Udayanga et al., 2018; Dong et al., 2015; He et al., 2010; Kistler et al., 1987). Based on the assumption that these metals are retained, shifts in the mobile heavy metal fractions between FS and char were evaluated.

## 3 Results and discussion

### 3.1 Feedstock characterisation

The feedstock characteristics are summarised in Table 2. Ultimate and proximate analysis showed higher levels of C, H, N, volatile matter and fixed carbon in N-FS. This was attributed to a higher ash content in W-FS compared to N-FS (47.3% and 25.1%, respectively), also leading to a lower calorific value (18.3 and 12.3 MJ/kg, respectively). High ash contents in FS as in Warangal are often associated with grit and sand originating from poorly lined containment structures (Niwagaba et al., 2014).

**Table 2:** Characterisation of feedstock material.

Parameter	Unit	N-FS	W-FS	PF
Volatile matter	[%]	63.7	47.4	77.3
Fixed carbon	[%]	11.2	5.3	14.4
Ash	[%]	25.1	47.3	8.3
C	[% w/w]	41.11	28.42	44.85
H	[% w/w]	4.95	3.40	5.58
N	[% w/w]	4.36	2.55	0.58
S	[% w/w]	1.57	1.66	1.11
P	[g/kg]	0.81	1.54	0.01
Ca	[g/kg]	32.86	56.68	5.40
Mg	[g/kg]	4.28	4.84	0.93
K	[g/kg]	1.58	2.63	5.16
HHV	[MJ/kg]	18.3	12.3	17.6

Phosphorus (P) concentrations in W-FS and N-FS (1.54 and 0.81 g/kg, respectively) were lower than in previous reports for FS, e.g. 31 and 24 g/kg in China and Uganda, respectively (Liu et al., 2014; Gold et al., 2017). FS from septic tanks may have significantly lower P concentrations as P is largely not retained in the septic tank sludge. Septic tanks, only separating the solid from the liquid fraction (Cairncross and Feachem, 1993), discharge soluble P. Only 20–30% of total P is expected to settle (Lusk et al., 2017). Further, this organically bound and condensed P is hydrolysed over time to form soluble orthophosphate leaving in the effluent (Wilhelm et al., 1994; Gill et al., 2009).

**Fig. 3:**
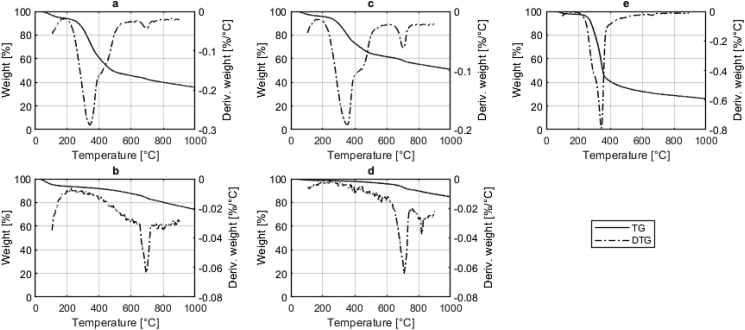
TG and DTG results from N-FS (a), N-CBC (b), W-FS (c), W-CBC (d) and PF (e).

The results from thermal analysis for PF, W-FS and N-FS are shown in Fig. 3. Any weight losses below 180^∘^C seen for FS and PF are caused by drying and dehydration reactions (Magdziarz and Werle, 2014). PF shows the typical weight loss between 150 and 500^∘^C caused by the decomposition of hemicellulose, cellulose and lignin (Basu, 2010) while the weight loss for FS is also associated with the decomposition of carbohydrates, protein and fats (Ward et al., 2014; Liu et al., 2014). Both PF and FS reach maximum weight loss rates between 340 and 350^∘^C. The cessation of weight loss from approximately 500^∘^C onwards for FS and 400^∘^C for PF indicates that the main pyrolysis reactions are complete. From an energy standpoint this suggests that operating under conditions that expose the feedstock particles to 500^∘^C would be sufficient for biochar production. However, the whole system must be considered to ensure enough heat is generated for thermal drying of the feedstock.

A further weight loss peak between 650 and 750^∘^C was observed for the FS, which has also been reported for sewage sludge and has been attributed to the decomposition of carbonates (Kwon et al., 2018). In this temperature range calcium and magnesium carbonates decompose from CaCO_3_ and MgCO_3_ to CaO and MgO respectively. The CO_2_ formed in these reactions causes the observed weight loss.

### 3.2 Solid fuel characteristics

Among many other parameters the solid fuel characteristics of W-BC and N-BC are given in Table 3. Higher average HHVs of N-BC compared to W-BC are associated with a lower ash content in the char, resulting from the lower ash content of the precursor material. The HHVs reported here are in agreement with those reported for FS-derived chars produced at temperatures between 450 and 750^∘^C, being 8.8–17.91 MJ/kg (Gold et al., 2018; Ward et al., 2014). A comparison with the control samples shows that the omission of PF with its high fixed carbon and low ash content of 14.4% and 8.3%, respectively, decreased the derived char HHVs by 6.7% and 49.5% between N-BC and N-CBC and between W-BC and W-CBC, respectively. This finding supports recommendations to blend FS with other biomass before pyrolysis to lower the ash content improving the fuel quality of the derived chars (Andriessen et al., 2019).

**Table 3:** Physical and chemical biochar properties, comparative literature values and international guidelines.

Parameter	Unit	N-BC	N-CBC	W-BC	W-CBC	Uganda^a^	Ethiopia^b^	IBI^c^	EBC^d^	EU^e^	USEPA^f^
Sample size		12	1	12	1						
Volatile matter	[%]	20.3 ± 4.0	21.1	14.2 ± 2.5	13.3	6.7–26.1					
Fixed carbon	[%]	34.1 ± 3.9	28.6	17.2 ± 5.2	6.1	18.8–23.3					
Ash	[%]	45.6 ± 4.2	50.3	60.8 ± 5.5	80.6	54.5–73.8					
HHV	[MJ/kg]	14.9 ± 0.9	13.9	9.7 ± 1.5	4.9	8.8–12.4					
pH	[ ]	10.5 ± 0.5	9.4	10.8 ± 1.2	12	9.1–11.2	8.23				
CaCO_3_-equ.	[%]	13.8 ± 2.7	16.9	20.1 ± 2.8	23.7						
Ca	[g/kg]	56.4 ± 3.9	63.3	89.4 ± 11.5	103.4		32.8				
Mg	[g/kg]	7.8 ± 0.7	8.9	9.6 ± 1.7	12		28.9				
K total	[g/kg]	8.1 ± 0.8	8.1	11.7 ± 1.9	8.8		8.21				
K available	[% of total]	77.7 ± 2.9	78.3	71.9 ± 9.4	58.1						
P total	[g/kg]	1.2 ± 0.2	1.7	2.2 ± 0.6	2.2	31–42	42.7				
P available	[% of total]	61.0 ± 6.4	52.1	53.7 ± 12.1	41.5	75.3–98.3					
Cd	[mg/kg]	13.5 ± 2.7	15.1	12.4 ± 2.0	15.5		1.23	1.4–39	1.5	20–40	85
Cr	[mg/kg]	56.1 ± 5.8	92	54.3 ± 16.5	98.6	113.7–194.2	39.5	93–1200	90		3000
Cu	[mg/kg]	463.0 ± 61.1	541.1	310.3 ± 37.0	370.7	81.8–113.2	214	143–6000	100	1000–1750	4300
Ni	[mg/kg]	122.7 ± 37.1	100.3	164.1 ± 48.8	184.9	57.4–96.5	84.4	47–420	50	300–400	420
Pb	[mg/kg]	395.3 ± 57.9	552.9	241.7 ± 50.9	311.9	<5–21.5	502	121–300	150	750–1200	840
Zn	[mg/kg]	1516.9 ± 209.1	2173.2	1072.9 ± 326.9	1385.2	872.9–1116	28400	416–7400	400	2500–4000	7500

a (Gold et al., 2018).

b (Woldetsadik et al., 2016).

c Maximum thresholds for biochar (IBI, 2015).

d Basic quality biochar (EBC, 2019).

e Limit values in sludge for use in agriculture (EEC, 1986).

f Ceiling concentrations for biosolids applied to land (Walker et al., 1994).

The high average volatile matter of 20.3% in Narsapur and 14.2% in Warangal can be explained by three factors evident from the TGA and DTG plots of W-CBC and N-CBC. Firstly, non-completion of the main pyrolysis reactions causes residual weight loss between 150 and 500^∘^C. Secondly, carbonates are only partially decomposed in the process, as seen by the DTG peak between 650 and 750^∘^C. Thirdly, continued heating from 750^∘^C to 1000^∘^C as part of proximate analysis causes further continuous weight loss due to ongoing carbonisation at temperatures higher than those reached in the treatment process.

Experience shows energy recovery from FS to be more profitable than the production of soil amendments (Diener et al., 2014). Dried FS in Uganda and Senegal with similar calorific values to N-BC and W-BC (10.9–13.4 MJ/kg) was deemed suitable as an industrial fuel for brick kilns (Gold et al., 2017) suggesting a similar use for these chars. However, the most suitable end use of the FS-derived products will depend on local economic and social conditions and should be based upon market studies (Reymond, 2014).

### 3.3 Agronomic value

**Fig. 4:**
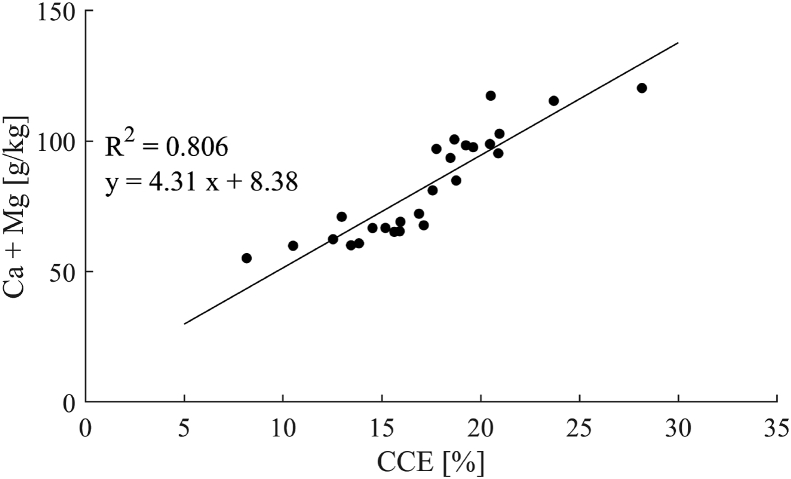
Relationship between CCE and cumulative Ca and Mg concentrations of all studied biochars.

Both N-BC and W-BC had alkaline pH values averaging 10.5 and 10.8, respectively. Biochars typically show an alkaline pH, as the fraction of inorganic elements increases in pyrolysis relative to the feedstock (Novak et al., 2009) and as acidic functional groups on the material’s surface are lost (Mukherjee et al., 2011). While these values indicate that the char might be suitable for liming acidic soils, a more quantifiable method is the assessment of calcium carbonate equivalents (CCE) (Ippolito et al., 2015). Average CCE values of 13.8% and 20.1% were found for N-BC and W-BC, respectively. They lie in a similar range to the liming potential of biochars from other excreta-derived feedstock including sewage sludge, cattle and poultry manure (Wang et al., 2012; Singh et al., 2017). The high liming potential is associated with high concentrations of Ca and Mg. Accordingly, high cumulative concentrations of 72.2 and 115.4 g/kg were found for N-CBC and W-CBC, respectively, and positive correlation ($R^{2}$=0.806) between CCE and cumulative Ca and Mg concentrations (Fig. 4). The presence of carbonates in the biochars observed by TGA may indicate that carbonate species were only partially thermally degraded to Ca and Mg oxides. However, it is likely that the carbonates were reformed upon cooling. Exposure to moisture and CO_2_ cause Ca and Mg oxides to react to hydroxides and carbonates (Housecroft and Sharpe, 2012). The speciation of Ca and Mg is of subordinate importance to the product quality of the biochars. Their carbonates, oxides and hydroxides may all be used for liming purposes as they all react with water and CO_2_ to form the bicarbonates that help neutralise acidic soil conditions (Brady and Weil, 2008).

Nutrient concentrations of 1.2gP/kg and 8.1gK/kg were measured for N-BC and 2.2gP/kg and 11.7gK/kg for W-BC. There remains a paucity of published data on the nutrient content of FS-derived biochars. As discussed for the feedstock, the derived biochars also show considerably lower P concentrations compared to other FS-derived biochars which are typically between 31 and 81gP/kg (Gold et al., 2018; Liu et al., 2014; Woldetsadik et al., 2018). In this case study, the process is limited by the quality of the feedstock. Feeding material with higher P concentrations, e.g. as reported in Ghana (Nartey et al., 2017; Cofie et al., 2009), would improve the fertilising value of the derived chars. Compared to P, the K concentrations were in closer agreement to literature values between 19 and 29gK/kg (Woldetsadik et al., 2018; Liu et al., 2014). However, the higher K concentrations in the PF compared to N-FS and W-FS indicate that the addition of PF improved the fertilising value of the resulting biochar. A comparison between the control samples and those with addition of PF shows that all elements associated with the ash fraction (Ca, Mg, K, P) increase in the control sample except for K. Potassium concentrations remain equal for N-BC and N-CBC and drop from W-BC to W-CBC, confirming the benefit of co-feeding PF to produce biochar for fertilising purposes.

P_*available*_ and K_*available*_ as a fraction of total concentrations were between 71.9 and 77.7% for K and 53.7–61.0% for P. This partial availability of P, caused by the transformation into more stable forms through pyrolysis, has been deemed beneficial to agriculture, as P is released over longer periods of time (Dai et al., 2016). Both pyrolysis temperature and biomass addition will influence P availability (Zwetsloot et al., 2015), but the precise mechanisms of P transformation and consequences for P availability are still uncertain (Wang et al., 2012). For many biochars, available K is equivalent to total K (Schmidt et al., 2015), which was not confirmed for FS-derived biochars. It has been suggested that entrapment of K into the carbon structure as well as bonding into more stable forms causes this immobilisation (Kong et al., 2014).

Heavy metals in the feedstock are concentrated in the ash fraction of the derived biochars (Beesley et al., 2015; Caballero et al., 1997). The origin of heavy metals in FS, besides the excretion through humans, include industrial effluents, leachate infiltration from solid waste dumps or illegal disposal of hazardous materials such as batteries to latrines (Appiah-Effah et al., 2015). The heavy metal concentrations found in this study are summarised in Table 3. All heavy metal concentrations were within the ceiling concentration limits set out by the USEPA (Walker et al., 1994) and the limit values set by the EU (EEC, 1986) for land application of sludge. Apart from Pb in N-BC, W-BC and N-BC also comply with the biochar specific guidelines issued by the International Biochar Initiative (IBI, 2015). The chars did not comply with the stricter limits for biochar of the European Biochar Initiative (EBC, 2019). Concentrations of Zn, Pb, Ni and Cu in N-BC and W-BC all breached EBC limits.

Besides heavy metal contamination, pathogenic contamination is a main concern when recovering resources from FS (Kengne et al., 2014), especially in agriculture. Due to the high process temperatures, pyrolysis is known to eliminate any pathogens within seconds (Liu et al., 2014). Therefore, as in previous FS pyrolysis studies (Woldetsadik et al., 2018; Gold et al., 2018; Ward et al., 2014), full pathogen destruction can be assumed.

### 3.4 Heavy metal mobility

With respect to heavy metal concentrations, notable variation was observed, with relative standard deviations ranging between 10.3 and 30.5% in N-BC and W-BC. Given that FS is fed to the process in batches and the holding tanks allow for only limited homogenisation, the batch delivery is likely to be one cause of these variations. In addition, the ratios of co-treated PF are approximate.

**Fig. 5:**
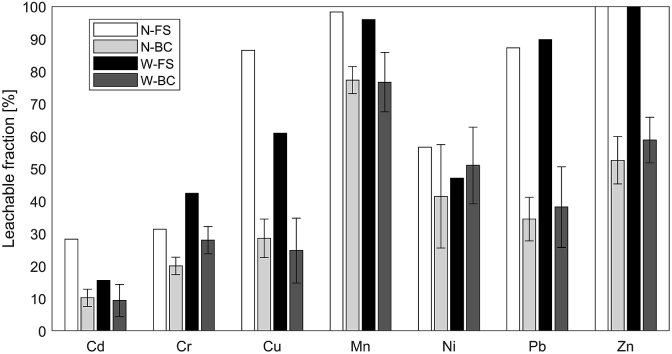
Mobility of heavy metals in FS and their derived biochars. Error bars denote standard deviations.

Regarding the heavy metal concentrations, it should be considered that their bioavailability is influenced by pyrolysis. Sequential extractions of sewage sludge biochars showed that the majority of heavy metals were transformed from exchangeable and acid-leachable forms to non-bioavailable forms (Jin et al., 2016). Fig. 5 compares the leachable fractions of heavy metals between FS and biochar to evaluate their mobility. Mobility is expressed as the fraction of the HCl-extractable and acid-digestible concentrations. Analysis showed, for example, that Cd, Cr and Cu were 10.4%, 20.0% and 28.1% leachable from N-BC and 8.9%, 27.1% and 24.6% leachable from W-BC, respectively. Except for Ni, the mobility of all heavy metals was higher in the FS compared to the derived biochars, as shown in Fig. 5 (e.g. Cu leachability dropped from 86.5% in N-FS to 28.6% in N-BC).

The comparison between FS and control samples avoids possible contamination introduced by the PF. Table 4 shows the shifts in mobility of non-labile heavy metals induced by the treatment process. Leachable fractions >100% for Zn are likely to be attributable to losses during the dry-ashing step preceding acid digestion. Immobilisation is evident for all non-labile heavy metals apart from Ni. The highest reductions in mobility were achieved for Cu, Cr and Zn, which dropped by 51.2%, 59.2%,and 59.6% in Narsapur and by 55.0%, 65.2% and 48.6% in Warangal, respectively. Again, no immobilisation was observed for Ni, confirming the observations for N-BC and W-BC. These results agree with reductions of Cu, Pb and Zn mobility through pyrolysis reported for contaminated soil (Wijesekara et al., 2007). Further, the addition of PF may have enhanced immobilisation. Co-pyrolysis of contaminated soil with woody biomass was shown to decrease heavy metal mobility depending on process temperature, type of heavy metal and extent of biomass addition (Debela et al., 2012). This was attributed to encapsulation within the biochar structure.

**Table 4:** Comparison of leachable heavy metals fractions between FS and control samples.

Parameter	Unit	N-FS	N-CBC	W-FS	W-CBC
Cr	[%]	31.4	12.8	42.4	19.1
Cu	[%]	86.5	42.2	60.9	21.2
Mn	[%]	98.3	74.4	96.0	73.9
Ni	[%]	56.6	54.6	47.1	50.9
Zn	[%]	101.4	41.0	103.5	53.2

### 3.5 Particle size distribution and structural analysis

**Fig. 6:**
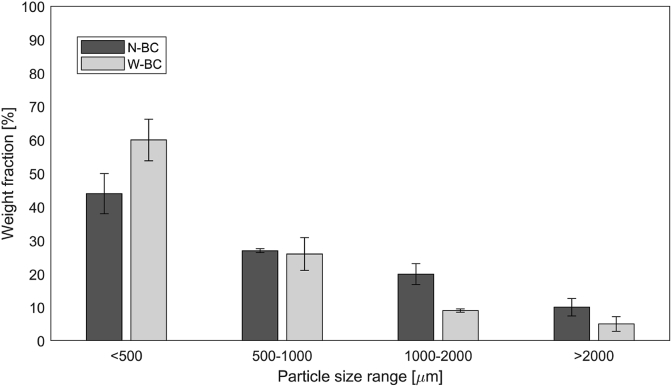
Particle size distribution of N-BC and W-BC. Error bars denote standard deviations.

The particle size distribution of N-BC and W-BC showed the largest particle fraction to be < 500 μm, namely 43.8% and 60.0%, respectively (see Fig. 6). Using the classification put forward by Schmidt et al. (2015), N-BC can be described as blended powder, W-BC as fine powder. Particle size will be influenced not only by feedstock characteristics, but also by the process itself (Cetin et al., 2004). High heating rates in particular, given in fast pyrolysis, produce fine biochars (Bruun et al., 2012). The combination of the high heating rates within a continuous reactor and the particulate nature of dried FS are likely to be responsible for the resulting fine particle classes. Additionally, the handling mechanisms of the process contribute to a smaller particle size, such as auger feeds breaking up particles.

**Fig. 7:**
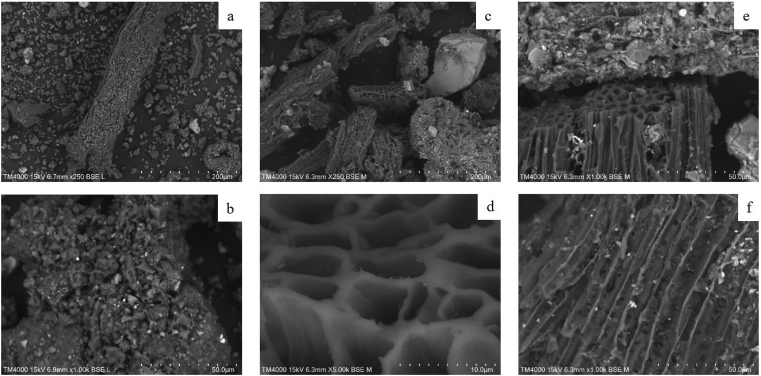
SEM images of N-FS (a,b), N-BC (c,d), W-BC (e,f).

Fig. 7 shows SEM images of FS and biochars. The compositional contrast causing elements heavier than C to appear lighter (Singh et al., 2017) allows for differentiation between mineral and organic material. The FS samples show agglomerates of carbonaceous material devoid of macroporous structures. Mineral constituents are covered with, or incorporated into, the organic matter. In N-BC and W-BC volatile matter has been thermally degraded. Besides larger grains of sand that are now visible, mineral matter is also shown to be attached to the surface of carbonaceous material and to be trapped in the carbonaceous structures. Fig. 7e indicates that sintering of the material has occurred, where mineral components are welded together into a solid aggregate. Sintering was clearly observable in the full-scale process: sintered mineral depositions had to be cleared from the reactor on a weekly basis.

The decomposition of the volatile matter leaves behind macroporous structures that are evident for both N-BC and W-BC. These macropores of a biochar are derived from the cellular structure of the precursor material (Wildman and Derbyshire, 1991) and can be seen in cell wall structures preserved in the biochar (see Fig. 7d). Fig. 7f shows that in parts the cell wall structures have been damaged, they are permeated by holes. This could be explained by the nature of the reactor which admits a limited amount of oxygen to sustain the heat demand of the process. The perforated structure suggests that the cell walls were partially oxidised which would have otherwise remained intact in an oxygen-free environment.

The N-CBC and W-CBC also showed cellular macroporous structures, indicating that these not only originate from the added PF but also from undigested fibrous food matter. This suggests that higher amounts of fibrous food matter in FS lead to more macroporous structures in the derived biochars. The composition of faeces is significantly influenced by the intake of indigestible fibre, which is higher for vegetarian and low-income country diets (Rose et al., 2015). Andhra Pradesh has among the smallest fraction of vegetarians (1.75%) of all the Indian states. Yet this may suggest FS-derived biochars to be more macroporous in states with a higher vegetarian population, e.g. Rajasthan, Punjab or Madhya Pradesh at 74.9%, 66.75% and 50.6%, respectively (Statista, 2016).

Biochar has been discussed as a soil amendment improving the physical properties of soil such as the water-holding capacity (Ippolito et al., 2015). The main mechanisms driving this improvement are the porous structures inherent to biochar and irregular biochar shapes causing increased void space between soil particles (Liu et al., 2017). Therefore, soil physical properties are likely to benefit from the macroporosity found in N-BC and W-BC. Larger spaces between soil particles in sandy soils cause leaching losses (Eash et al., 2016). The fine nature of these biochars could increase the water retention of such soils by decreasing void space between soil particles.

## 4 Conclusions

This study assessed and compared the biochar produced by full-scale pyrolytic FS treatment at two sites in India. Co-treatment of FS with agricultural waste yielded macroporous, powdery biochars whose physical and chemical properties suggest potential applications as solid fuel or soil amendment media. The key findings of this study were:

• Calorific values were limited by high ash contents, but average values as high as 14.9 MJ/kg in Narsapur suggest potential for use as a solid fuel for energy recovery.
• A high liming potential, especially for biochars from Warangal of 20.1% CCE, indicate suitability to alleviate acidic soil conditions.
• Biochar nutrient concentrations were restricted by the nutrient-depleted nature of septic tank FS.
• Heavy metals present in FS were concentrated in the biochar. However, their mobility was considerably reduced, especially in the case of Cu and Zn, where mobility dropped by 51.2–65.2% and 48.6–59.6%, respectively.
• Co-processing of FS with other carbon rich waste streams can be used to influence desired biochar properties such as nutrient enrichment and fixed carbon concentrations, as well as providing an additional source of energy.

Biochar production for FS treatment has been demonstrated at scale and is now being adopted by an increasing number of municipalities in India. This study reports for the first time, a comprehensive study of the properties of FS biochar made using a commercial scale system and has provided preliminary evidence informing the end use of FS-derived chars. Moving forward, research should focus on possible organic contaminants in FS-derived biochars, its practical application as a solid fuel and its performance in crop-growing trials.

## Declaration of competing interest

The authors declare that they have no known competing financial interests or personal relationships that could have appeared to influence the work reported in this paper.
